# 

**Published:** 1826-01

**Authors:** 


### THE MEDICO-CHIRURGICAL REVIEW.

*NEW SERIES.*

---

### VOLUME FOUR.

---

[BEING VOL. VIII. OF ANALYTICAL SERIES.]

CONDUCTED BY

ASSOCIATED PHYSICIANS AND SURGEONS;

AND SUPERINTENDED BY

JAMES JOHNSON, M.D.

MEMBER OF THE ROYAL COLLEGE OF PHYSICIANS OF LONDON, AND PHYSICIAN EXTRAORDINARY TO HIS ROYAL HIGHNESS THE DUKE OF CLARENCE.

---

Nec tibi quid liceat sed quid fecisse decebit
Occurrat mentemque domat respectus honesti.—CLAUD.

---

Surgeon Genl's Office LIBRARY 5744 Washington D.C.

LONDON:

*Printed by G. Hayden, Little College Street, Westminster.*

Published by Burgess & Hill, Windmill Street, Haymarket; Baldwin, Cradock, and Joy, Pater-Noster Row; Kingsbury, Parbury, & Allen, Leadenhall Street; Highley, and T. & G. Underwood, Fleet Street; Callow & Wilson, Princes Street, Soho; Cox & Son, Borough; Jackson, Borough; Anderson, West Smithfield; John Cox, Berners Street, Oxford Street; Adam [illegible]ack, Edinburgh; D. Lizars, Princes Street, Edinburgh; Richard Griffin & Co., W. R. M'Phun, and David Allan & Co., Glasgow; Hodges & M'Arthur, Dublin; Messrs. Treuttel & Wurtz, Paris and Strasburg.

---

1826.

1. THE LONDON DISPENSATORY; containing—1. The Elements of Pharmacy—2. The Botanical Description, Natural History, Chemical Analysis, and Medicinal Properties, of the Substances of the Materia Medica—3. The Pharmaceutical Preparations and Compositions of the latest editions of the Pharmacopœias of the London, Edinburgh, and Dublin Colleges of Physicians. The whole forming a practical Synopsis of Materia Medica, Pharmacy and Therapeutics; illustrated with many useful Tables and Plates of Pharmaceutical Apparatus, and Synonyma of the names of the articles of the Materia Medica, and the Pharmaceutical Preparations in almost every spoken language. By ANTHONY TODD THOMPSON, M.D. F.L.S. In 1 large Vol. 8vo. of upwards of 1000 pages, 15s. Bds. greatly improved, a 4th Edition.

2. A PRACTICAL SYNOPSIS of CUTANEOUS DISEASES, according to the arrangement of Dr. WILLAN, exhibiting a concise View of the Diagnostic Symptoms, and the Method of Treatment. By THOMAS BATEMAN, M.D. F.L.S. late Physician to the Public Dispensary, and to the Fever Institution. In 8vo. price 12s. Bds. (with a Plate of the Eight Orders, beautifully coloured) the 6th Edition.

*By the same Author,*

3. DELINEATIONS of the CUTANEOUS DISEASES, comprised in the Classification of the late Dr. Willan. In 4to. with upwards of 70 coloured Plates. Price 12*l.* 12s. Bds.

The Series of new Engravings, representing those Diseases which should have been figured in the subsequent Parts of Dr. Willan's unfinished Work, may be had by the Possessors of that Work, separate, price 7*l.* Bds.

4. SURGICAL OBSERVATIONS on the Constitutional Origin and Treatment of Local Diseases; and on Aneurisms; including Directions for the Treatment of Disorders of the Digestive Organs. By JOHN ABERNETHY, F.R.S. Surgeon to St. Bartholomew's and Christ's Hospitals, &c. &c. In 8vo. price 8s. bds. a new Edition.

*By the same Author.*

5. On DISEASES resembling SYPHILIS, and on the URETHRA, 6s.

6. On INJURIES of the HEAD, and MISCELLANEOUS SUBJECTS, 7s.

7. On LUMBAR ABSCESSES and TUMOURS, 6s.

The whole of the above may be had in 2 Vols. 8vo. 1*l.* 7s. bds.

8. PHYSIOLOGICAL LECTURES, addressed to the College of Surgeons, complete in 1 Vol. 8vo. 18s. bds.

9. On GALL and SPURZHEIM'S PHYSIOGNOMY, &c. price 3s.

10. ADDITIONAL OBSERVATIONS on the TREATMENT of certain severe Forms of HEMORRHOIDAL EXCRESCENCE, illustrated by Cases; with the History of a Case in which an enlarged Parotid Gland was successively removed. By JOHN KIRBY, A.B. lately President of the Royal College of Surgeons in Ireland, &c. &c. &c.

11. THOUGHTS on MEDICAL EDUCATION, and a Plan for its Improvement; addressed to the Council of the University of London. In 8vo. Stitched. Price 2s.

### XIV.

### BIBLIOGRAPHICAL RECORD;

OR,

*Works received between the 15th of September, and the 15th of December, 1825.*

1. Thoughts on Vaccination, and the Causes of its failing to afford the same Protection against Variola as formerly. By JOHN M'GHIE, Surgeon, R.N. &c. &c. Octavo, sewed, pp. 38. Dumfries, September 20th, 1825.

☞ *The cause of diminished protection is supposed, by our author, to be some change which the vaccine virus has undergone, by passing through a succession of human subjects. This supposition had been advanced long before Mr. M'Ghie's paper appeared, but, to this day, there is nothing like a proof of its truth produced. It is, therefore, needless for us to go into the subject here.*

---

2. L'Ouie et la Parole rendues à Honoré Trezel, sourd-muet de naissance, precedé d'un Rapport fait à l'Academie des Sciences. Par le Docteur DELEAU, Jeune, M.D. &c. Octavo, pp. 52. Paris, September, 1825.

☞ *By the kindness of Dr. Edwards.*

---

3. Revue Medicale, Françoise et Etrangère, et Journal de Clinique de l'Hôtel Dieu et de la Charité, Aout, Septembre, et Octobre, 1824.

*In Exchange.*

---

4. Report of the Committee on Laws, to the Corporation of the City of New York, on the subject of Interment within the populous Parts of the City. Read and adopted at a Special Meeting of the said Corporation, on the 9th June, 1825. New York, July, 1825.

☞ *A sharp controversy exists, on the West of the Atlantic, respecting the salubrity or insalubrity of the custom of burying the dead in private vaults, churches, church-yards, &c. within the City. The Profession, as usual, is divided, and a long list is published, in favour of the innocuity of the intra-urbal sepulture. On every account we think the city of the dead should be separated from the city of the living, and, therefore, we should vote for extra-urbal sepulture. We cannot, however, go into the arguments pro and con.*

---

5. A short Illustration of the Advantages derived by the use of Sulphureous, Fumigating, hot Air, and Vapour Baths, in a variety of obstinate Diseases. By JONATHAN GREEN, late Surgeon in His Majesty's Navy, No. 5, Bury-street, St. James's. Octavo, sewed, pp. 40. Price 1s.

---

6. An Essay on Egyptian Mummies; with Observations on the Art of Embalming among the ancient Egyptians. By A. B. GRANVILLE, M.D. F.R.S. &c. &c. &c. Quarto, pp. 49, with Plates. [From the Philosophical Transactions.]

7. An Introduction to the Use of the Stethoscope: with its Application to the Diagnosis in Diseases of the Thoracic Viscera; including the Pathology of these various Affections. By WILLIAM STOKES, M.D. Octavo, pp. 224. Edinburgh, September, 1824.

☞ *To the student of auscultation, (and the whole of the rising generation must study it) this little work offers an improved plan, and, consequently, an increased facility. Dr. Stokes selects and combines judiciously from publications of Laennec, Forbes, Andral, Collin, and others. This book forms a medium between the voluminous work of Laennec, and the compressed pocket compilation of Collin. We can recommend it with confidence.*

---

8. Lectures and Observations on Medicine. By the late MATTHEW BAILLIE, M.D. Royal octavo, pp. 242. London, 1825.

☞ *By the will of the late lamented Dr. Baillie, these posthumous papers were printed, but not published. His words were—" one hundred and fifty copies may be printed, of which one copy may be given to each of my more intimate friends, and the remainder to the Royal College of Physicians." We are proud to be numbered among his friends. We shall, of course, give a full account of these posthumous papers, which the amiable author was too modest to publish to the world in a formal shape.*

---

9. Sketches of the most prevalent Diseases of India; comprising a Treatise on the Epidemic Cholera of the East; Statistical and Topographical Reports of the Diseases in the different divisions of the Army under the Madras Presidency; embracing, also, the Annual rate of Mortality, &c. of European Troops; and practical Observations on the effects of Calomel on the Alimentary Canal, and on the Diseases most prevalent in India. By JAMES ANNESLEY, Esq. Madras Medical Establishment; lately in charge of the General Hospital, Madras, and Garrison Surgeon of Fort St. George. 8vo pp. 464, three beautifully coloured plates. Price 18s. boards. Underwoods, October, 1825.

☞ *Of this very valuable work we shall give a full account as early as possible.*

---

10. Elements of the Anatomy of the Human Body, in its sound state; with occasional remarks on Physiology, Pathology, and Surgery. By ALEXANDER MONRO, M.D. F.R.S.E. Fellow of the Royal College of Physicians, and Professor of Medicine, Anatomy, and Surgery, in the University of Edinburgh. Illustrated by twelve engravings. Vols. 2, 8vo. pp. 602, and pp. 617. Maclaghlan and Stewart, Edinburgh, October, 1825.

☞ *For an account of this valuable work see the present number, page* 52, *et seq.*

---

11. The Philadelphia Journal of the Medical and Physical Sciences. Edited by Drs. CHAPMAN, DEWEES, and GODMAN. No. 2, New Series. Philadelphia, 1825. *(In exchange.)*

---

12. Useful Hints to Travellers, going to, or already arrived in, South America; and to Military Men, or Merchants, bound to the West Indies, India, or any other Tropical Climate. London, 12mo. pp. 120. J. Churchill, 1825.

☞ *The selection is rather judicious, and this little work may prove a useful pocket-companion to the Traveller in foreign, and particularly inter-tropical climates. There are some errors respecting the doses of medicines, which should be corrected.*

13. An Address to the Members of the Royal College of Surgeons of London, on the injurious conduct and defective state of that Corporation; with reference to Professional Rights, Medical Science, and the Public Health. By JOHN ARMSTRONG, M.D. Lecturer on the Principles and Practice of Physic, and Consulting Physician to the London Fever Hospital. 8vo. sewed, pp. 48. October, 1825.

☞ *Vide Medical Jurisprudence in this Number, page* 273, *et seq.*

---

14. A Century of Surgeons on Gonorrhœa, and on Strictures of the Urethra. "*Multum in parvo.*" Small, 8vo. pp. 184. Whittaker, 1825. Price 7s.

☞ *Dr. Kitchener has turned his attention from Cookery to Clap-curing, (a curious transition) and, although, we do not like his physic so well as his music, the* fruit *of his present lucubrations will be no bad* desert *for the student, though a pretty dear one (seven shillings for* 184 *pages of pocket octavo!!).*

---

15. Revue Medicale, Françoise et Etrangère, et Journal de Clinique, de l'Hôtel Dieu, et de La Charité de Paris, for September, October, and November, 1825.

---

16. Report of the Weekly Board, respecting certain Charges brought against St. George's Hospital. 8vo, sewed, pp. 40. October, 1825.

☞ *We have already alluded to this subject in a former Number, and need not again take it up. The whole was only a nine day's wonder, and is now forgotten. We never believed the calumnies which were circulated on the occasion, and needed no formal refutation of them. It was proper, perhaps, to have recourse to this measure on account of the Governors of the Institution and the public in general.*

---

17. New York Medical and Physical Journal. Nos. 13, and 14. New York, January and April, 1825. *(In exchange.)*

---

18. An Address to the Inhabitants of Lancashire, and of the Adjoining Counties, on the Present State of the Medical Profession, with Remarks on the Elementary Education of the Student, &c. &c. By THOMAS TURNER, Member of the Royal College of Surgeons, Lecturer on Anatomy, &c. 8vo. sewed, pp. 26. 1825.

☞ *The object of this well-written little pamphlet is intended to shew the practicability and importance of establishing a school (on a more extended scale) in Manchester, for the cultivation of Medical and Surgical science. The observations and arguments of Mr. Turner are well worthy of the attention of those to whom they are addressed.*

---

19. Disputatio Medica inauguralis de Morbis Syphiloideis, vel Pseudosyphiliticis. Auct. THO. HARRISON BURDER, M.D. Edinburgi, 1815.

☞ *Of this most valuable and practical Thesis, we gave a translation in an early number of the Medico-chirurgical Journal, above nine years ago. It is, in fact, one of the best tracts on the subject which we have ever read.*

---

20. An Essay on the Nature, Causes, and Treatment of Water in the Brain. By WILLIAM SHEARMAN, M.D. and Physician to the Royal West London Infirmary. 8vo. pp. 123. Underwoods, November, 1825.

21. Elements of Physiology. By K. A. Rudolphi, M.D. Professor of Medicine, and Member of the Royal Academy of Sciences of Berlin. Translated from the German, by William Dunbar How, M.D. In three volumes, vol. I. pp. 254. November, 1825.

☞ *This promises to be a most valuable work. We shall wait the completion before we state our sentiments in detail.*

---

22. Observations on Transfusion of Blood; with an Account of Two Cases of Uterine Hæmorrhage, in which that Operation has been recently performed with Success. By Charles Waller, M.R.C.S. &c. &c. 8vo, sewed, pp. 34. Jackson, Borough, November, 1825.

---

23. A Short Description of the Bones, together with their several connexions with each other, and with the Muscles. By John South, Lecturer on Anatomy at St. Thomas's Hospital. Duodecimo, pp. 139. Jackson, Borough, November, 1825.

---

24. Additional Observations on the Treatment of certain severe forms of Hæmorrhoidal Excrescence, illustrated by Cases; with the History of a Case in which an enlarged Parotid Gland was successfully removed. By John Kirby, A.B. lately President of the Royal College of Surgeons in Ireland; one of the Surgeons to the Coombe Hospital, &c. 8vo. pp. 145. Dublin, Hodges and M'Arthur, 1825.

---

25. Dissertatio Medica Inauguralis de Synocha, sive Febre Flava. Auct. Alexander Manson, M.D. Edinburgi, 1811.

☞ *This little thesis contains, though written* 15 *years ago, as good pathological principles as any of the latest essays on the scourge of Europeans in the West—Yellow Fever.*

---

26. A Practical Treatise on the Arterial System. Intended to illustrate the importance of studying the Anastomoses, in reference to the Rationale of the new operation for Aneurism; and the Surgical Treatment of Hæmorrhage, with original coloured Plans. By Thomas Turner, Member of the Royal College of Surgeons, Lecturer on Anatomy, &c. 8vo. pp. 194. London and Manchester, November, 1825.

☞ *Mr. Turner appears to have availed himself of the best information on the subjects discussed in his volume.*

*He is wrong, however, in making Mr. Abernethy the first to tie the carotid artery. The late Mr. Fleming, a naval surgeon, performed the operation successfully, on board the Tonnant, of* 80 *guns, on the* 9*th of October,* 1803. *Dr. Coley, of Cheltenham, was assistant-surgeon of the ship at the time; and the case is in the Archives of the Naval Medical Board at this moment.—See Medico-chirurgical Journal for January,* 1817, *pp.* 2-3-4.

*We shall republish the Case next Quarter.—Ed.*

---

27. Observations on Tetanus; Illustrated by Cases, in which a new and successful mode of Treatment has been adopted. By Henry Ward, Surgeon. 4to. pp. 22. Gloucester, November, 1825.

☞ *This new practice is founded on two cases, successfully treated by large doses of the hydrocyanic acid—for instance, five drops every fifteen minutes till the violence of the spasms are allayed. We believe this medicine has been tried in tetanus before, both in this country and on the Continent.**

---

* *Dr. Trezevant, of Colombia, has detailed a case of this kind in the American Medical Recorder. It was unsuccessful.*

28. An Attempt to ascertain the Value of the Vaccine Virus, &c. By Felix Pascalis, M.D. &c. &c. of New York. 8vo. sewed, pp. 20.

Also, by the same Author,

An Examination of a Work, entitled Recherches Pratiques sur la Fièvre Jaune; Par A. S. Dariste, D. M. 8vo. sewed, pp. 5.

☞ *We beg to return our best thanks to Dr. Pascalis for these papers.*

---

29. A Manual of Anatomy, arranged so as to afford a concise and accurate Description of the Parts of the Human Body. From the French of A. L. J. Bayle. Revised and improved by William Bennett, M.D. 18mo. pp. 376, closely printed. Edinburgh, November, 1825. Price 7s. 6d.

☞ *This work, which is purely anatomical, and unmixed with physiological or surgical subjects, rests its claim on—" brevity, accuracy, superiority of arrangement, and great portability." It certainly is, as its motto expresses—" Multum in parvo."*

---

30. Medical Report of the Fever Hospital and House of Recovery, Cork-street, Dublin, for the Year 1825. With Observations on the Contagion of Plague and Typhus Fever. By Richard Grattan, M.D. &c. &c. &c. 8vo. sewed, pp. 54. November, 1825.

☞ *Dr. Grattan made a mistake in the title of this pamphlet. It should have been called a Critique rather than a Report. Not quite two pages of the work are on the subject of the Fever in the House of Recovery; the other 52 pages being criticisms on the writings of the anti-contagionists—especially Dr. Maclean, Mr. Larkin, Dr. Armstrong, and the Westminster Reviewer. We have neither time nor inclination to renew the discussion at present.*

---

31. The Institutes and Practice of Surgery; being the Outlines of a Course of Lectures. By William Gibson, M.D. Professor of Surgery in the University of Pennsylvania, &c. Volume the second, pp. 542, with plates. Philadelphia, 1825.

☞ *We have looked over this system of Surgery, which, though concise, we have observed to contain, not only the latest, but the best principles and practice of the art, conveyed in language well adapted to the subject, being at once terse and perspicuous.*

---

32. A Toxicological Chart, exhibiting, at one view, the Symptoms, Treatment, and modes of detecting the various Poisons, Mineral, Vegetable, and Animal, &c. 5th Edition. By William Stowe, Member of the Royal College of Surgeons. Price 2s. on two large sheets, folio. Anderson, November, 1825.

☞ *This edition is considerably enlarged and improved. It is by far the most complete thing of the kind which we possess.*

---

33. On Cholera, &c. By Thomas Brown, Surgeon Musselburgh. 8vo. sewed, pp. 56. 1824.

---

34. Remarks on some parts of Mr. Calvert's Treatise on Diseases of the Rectum; namely, Hæmorrhoids, Simple Stricture, and Spasmodic Constriction of the Sphincter Ani. By W. White, M.R.C.S. &c. of Bath. 8vo. pp. 79. Bath, November, 1825.

35. Researches into the Nature and Treatment of Dropsy in the Brain, Chest, Abdomen, Ovarium, and Skin; in which a more correct and consistent Pathology of these Diseases is attempted to be established, and a new and more successful Method of treating them recommended and explained. By JOSEPH AYRE, M.D. Member of the College of Physicians, &c. 8vo. pp. 242. London, December, 1825.

---

36. Medical Essays.—Essay first. On the Effects of Intestinal Irritation. Essay second. On some Effects of loss of Blood. Essay third. On Exhaustion and Sinking from various causes. By MARSHALL HALL, M.D. F.R.S.E. Physician to the General Hospital near Nottingham, &c. 8vo. pp. 96. London, December, 1825.

---

27. Further Observations on the Medicinal Leech; including a Reprint, from the Philosophical Transactions, of two Memoirs, comprising Observations on the Hirudo Vulgaris, or common Rivulet Leech; and on the H. Stagnalis and H. Complanata, now constituting the Genus Glossopora, with Illustrative Drawings. By JAMES RAWLINSON JOHNSON, M.D. F.R.S. F.L.S. &c. 8vo. pp. 112. London, December, 1825.

---

38. The Anatomy of the Fœtal Brain; with a comparative exposition of its structure in Animals. By FREDERICK TIEDEMANN, Professor of the University of Heidelburg, &c. Translated from the French of A. S. L. JOURDAN, by WILLIAM BENNETT, M.D. To which are added, some late Observations on the influence of the Sanguineous System over the development of the Nervous System in general. Illustrated by 14 plates. 8vo. pp. 324. Edinburgh and London, December, 1825. Price 12s. boards.

☞ *This is a most meritorious work, and ought to be in the hands of all Anatomists and Physiologists. We shall give some account of it as early as possible.*

---

IN THE PRESS.—We have received the first 15 sheets of a Work on "The Physical Treatment of Children." By Dr. DEWEES of Philadelphia. From a hasty glance over it, it appears to be a work of considerable merit, as might indeed be expected, from the talents and experience of the Author. We understand that Dr. Kennedy, of Glasgow, has a similar work (for Professional perusal) in the Press. We hope to review both productions in one article soon.

☞ *Dr. Dewees' work is publishing by Mr. Millar of Bridge-street, Blackfriars.*

---

*** NOTE.—Authors and publishers will readily appreciate the importance of having their works recorded, with full length titles, on this list, which stands as a perpetual advertisement so long as the Journal lasts, and as far as it extends. The republication of the Journal in America enhances the advantages of the BIBLIOGRAPHICAL RECORD, on which no work can be entered, unless transmitted, free of expence, to the Editor, under sealed cover to the publishers, or in any other way most convenient to the parties concerned.

*N. B.—As the Bibliographical Record closes on the* 15*th of the month preceding publication, all Works received after that date necessarily stand over till next quarter.*

### *Additional Subscribers since last Quarter.*

B.

Bland, Mr. Surgeon, His Majesty's Ship, Pyramus.

Bell, Dr. Demerara.

Bagster, Mr. Surgeon, Guilford Street, Russel-square.

Beresford, Mr. George, South America.

Borres, William, Esq. Surgeon, Dumferline.

Bythell, Richard, Esq. Member of the Royal College of Surgeons, St. Asaph.

Beatty, Dr. Molesworth Street, Dublin.

Broadbelt, James Lee, M.D.

Burney, Dr. R.N.

D.

Dix, Mr. William, Surgeon, Long Buckley, Northamptonshire.

De Witt, Mr. Surgeon, &c. Broad-street, City.

E.

Eves, Augustus, Esq. Member of the Royal College of Surgeons, and Licentiate of the Apothecaries' Company, Cheltenham.

G.

Gurr, John, Esq. Kilburn.

H.

Higginbottom, Mr. John, Member of the Royal College of Surgeons, Nottingham.

Huggins, Daniel, M.D. St. Vincents.

Hughes, Mr. Surgeon, &c. Shottisham.

J.

Johnstone, Dr. William, Hill-street, Edinburgh.

Jones, Richard Phillip, M.D. Physician to the Denby General Dispensary.

L.

Lizars, John, Esq. Surgeon, and Lecturer on Anatomy and Surgery, Edinburgh.

Ledgewick, Mr. M.R.C.S. Borobridge.

M.

Maclenern, Mr. R.N.

P.

Petch, Rev. Mr. Atherstone.

R.

Rodd, Mr. Surgeon, Hampstead.

Rutherford, George, Esq. to East Indies.

Rutter, Dr. John, Liverpool.

S.

Simpson, Mr. W. Surgeon, Hammersmith.

Sutherland, Mr. Surgeon Gerrard-street, Soho.

T.

Thomson, Mr. Surgeon, R.N.

Tritton, Mr. Surgeon, Broad-st. City.

Thomas, John, M.D. Physician at Cheltenham.

V.

Valentine, Mr. J. Somerton.

W.

Wilson, Mr. John, Student of Medicine, Edinburgh.

Wilde, G. R. Esq. Surgeon, Milden Hall, Suffolk.

